# Relationship Between Oxidative Stress in the Rotator Cuff and Transcutaneous Advanced Glycation End-Products Measurement in Diabetic Rats

**DOI:** 10.7759/cureus.67529

**Published:** 2024-08-22

**Authors:** Akinobu Kawada, Shingo Yoshitake, Ryuji Fujihara, Masakazu Ishikawa

**Affiliations:** 1 Department of Orthopaedic Surgery, Faculty of Medicine, Kagawa University, Takamatsu, JPN

**Keywords:** diabetes mellitus, rat model, oxidative stress, advanced glycation end-products, rotator cuff

## Abstract

Diabetes mellitus increases oxidative stress due to hyperglycemia, resulting in the degeneration of rotator cuff tissue. Currently, there is no established method to non-invasively assess the extent of this oxidative stress. To address this, we aimed to investigate the relationship between the accumulation of advanced glycation end-products (AGEs), a marker of oxidative stress, and transcutaneous autofluorescence intensity in rotator cuff tissue harvested from diabetic rats. Ten control Sprague-Dawley (SD) rats and streptozotocin-induced diabetic rats (n = 10 per group) were used. The rats were euthanized eight and 16 weeks after the induction of diabetes, and rotator cuff attachment sites were collected and histologically analyzed. Prior to euthanasia, autofluorescence intensity was measured transcutaneously in the rotator cuff area. The expressions of AGEs and type I collagen were evaluated immunohistochemically with specific antibodies and the stained areas were quantified. All data were statistically analyzed using the Mann-Whitney U test. Correlation analysis was performed for skin autofluorescence intensity and the percentage of AGEs staining area using Spearman’s rank correlation coefficient. The immunohistochemical expression of AGEs at the rotator cuff attachment sites and transcutaneous AGEs measurements were significantly higher in diabetic rats than in the control group at 16 weeks. There was no significant difference in the level of type 1 collagen between the two groups. This study reveals that the accumulation of AGEs in rotator cuff tissue increases due to prolonged hyperglycemia in diabetes. In addition, transcutaneous skin fluorescence intensity may be related to histological oxidative stress at the rotator cuff.

## Introduction

The incidence of rotator cuff tears in the shoulder increases with age, causing pain at night, muscle weakness, and a limited range of motion, and are associated with decreased Activities of Daily Living (ADL) and shoulder joint pain during daily activities [1,2]. The prevalence of rotator cuff tears is also known to increase in diabetic patients [3-5]. The mechanism of tendon degenerative change and subsequent injury in diabetes is thought to be influenced by the deposition of advanced glycation end-products (AGEs) due to oxidative stress in tissues [6,7]. Therefore, monitoring AGEs in tissues could enable the assessment of the pathological status of the rotator cuff and the risk of tearing.

Recent studies have shown that a non-invasive method of measuring AGEs, a transcutaneous fluorescence intensity measuring device, can be used to measure AGEs deposited in skin tissue, including the epithelium or dermis [8,9]. This non-invasive approach has the potential to predict the status of oxidative stress in individual organs. The accumulation of AGEs in skin tissue is associated with reduced skeletal muscle mass [10] and bone density [11] and may serve as a biomarker for lower back and lower extremity pain and numbness [12]. However, reports demonstrating the association between the accumulation of AGEs in skin tissue and the pathological conditions of tendon tissue, such as the rotator cuff, are scarce.

This study aims to investigate the correlation between non-invasively measured transcutaneous fluorescence intensity and histological AGEs accumulation in the rotator cuff in a diabetic rat model. We hypothesize that higher fluorescence intensity is associated with increased AGEs accumulation, which may have implications for assessing oxidative stress in rotator cuff tissue.

This article was previously presented as a poster at the ORS 2024 Annual Meeting on February 2-6, 2024.

## Materials and methods

Experimental animals

Nine-week-old male Sprague-Dawley (SD) rats (Crl: CD (cesarean derived)) were purchased from Charles River Laboratories Japan, Inc. (Yokohama, Japan). The rats were acclimatized under standard laboratory conditions with a 12-h light/dark cycle at 24±2°C. All animals had free access to commercial food (MF; Oriental Yeast Industry Co., Japan) and tap water. This study was conducted in accordance with the National Institutes of Health (NIH) guidelines for the use of laboratory animals, and the experimental protocol was approved by the author's affiliate, the Animal Care and Use Committee.

Experimental protocols

The rats were randomly divided into control (n = 10) and diabetic (n = 10) groups.

At 19 weeks of age, the diabetic group received a single intraperitoneal injection of streptozotocin (60 mg/kg; Fujifilm Wako Pure Chemical Corp., Ltd., Osaka, Japan) dissolved in citrate buffer (pH 4.0) [13]. The final concentration immediately before use was 30 mg/mL [14]. The control group received the same volume of citrate buffer intraperitoneally. All animals were handled with care to minimize stress, and any deviations in handling procedures were documented to ensure consistency across all experimental groups. Approximately 72 h after administration, blood glucose levels were measured from tail blood samples using a blood glucose meter (Accu-Chek Guide; Roche DC Japan KK, Tokyo, Japan), and rats with blood glucose levels exceeding 300 mg/dL were considered diabetic [15].

Approximately eight and 16 weeks after streptozotocin administration, five rats in each group were euthanized via the intraperitoneal administration of sodium pentobarbital and perfused transcardially with a fixative solution containing phosphate-buffered saline followed by 10% buffered formalin. The threshold of significance was set at p <0.05. After formalin fixation at 4°C for two days, the proximal humerus, scapula and rotator cuff were harvested in one lump. After demineralization and paraffin embedding, a 5-µm-thick thin-slice specimen of the anterior forehead, including the supraspinatus tendon attachment area, was prepared.

Transcutaneous AGEs fluorescence measurement

For transcutaneous AGEs measurements, as in previous reports, a device consisting of a light-emitting diode (LED) light source, a spectral device system with a 2048-pixel Charge Coupled Device (CCD) linear image sensor and grating, and a two-way silica-based optical fiber was used for each rat auricular estimated by measuring autofluorescence from the skin [16,17]. The device was calibrated before each measurement session using a standardized fluorescent reference to ensure accuracy. Calibration was conducted under controlled environmental conditions, with room temperature maintained at 24±2°C and ambient light minimized to reduce measurement variability. Measurements were taken consistently at the same time of day to account for potential circadian variations. Nine measurements were taken at eight and 16 weeks after streptozotocin administration prior to euthanasia, and the average values were used.

Immunohistochemical procedure

Sections were treated with 3% H2O2 for 10 min and normal horse serum for 30 min and incubated overnight at 4°C in the presence of primary antibodies against AGEs and type I collagen. Normal IgG was used as a negative control (Table 1).

**Table 1:** Summary of primary antibodies used in this study AGE: Advanced glycation end-products

Antibody	Source	Host	Concentration
AGEs	Abcam (ab23722)	Rabbit	10μg/mL
Collagen 1	Abcam (ab34710)	Rabbit	10μg/mL
Normal IgG	R&D systems (AB-105-C)	Rabbit	10μg/mL

Following the primary antibody incubation, sections were washed three times with phosphate-buffered saline (PBS) for 5 minutes each. The samples were then incubated with secondary antibodies (ImmPRESS Horseradish Peroxidase (HRP) Polymer Reagent Kit, Vector Laboratories, Burlingame, CA, USA) for 30 min at room temperature. After another set of three washes with PBS, followed by incubation with 3,3'-diaminobenzidine tetrahydrochloride and hydrogen peroxide (Nichirei Biosciences Inc., Tokyo, Japan) for 10 min at room temperature. Finally, the sections were stained with hematoxylin for 1 min.

Qualitative analysis of immunohistochemistry

The quantitative evaluation of AGEs accumulation in the rotator cuff attachment area was performed with sections stained for AGEs antigens using immunohistochemical staining and a microscope with a 10x objective lens (Olympus BX51, Tokyo, Japan). The region of interest (ROI) was the supraspinatus muscle tendon from the medial border to the attachment of the greater tubercle of the humeral head. In the ROI, stained areas were measured semi-automatically using WinROOF 2015 software (Mitani Corporation, Tokyo, Japan). The percentage of AGEs staining area (%) was obtained by measuring the area of the stained region using software and dividing it by the area of the ROI.

Statistical analysis

Statistical differences between groups were analyzed using the Mann-Whitney U test. Correlation analysis was performed for skin autofluorescence intensity and the percentage of AGEs staining area using Spearman’s rank correlation coefficient. All statistical analyses were performed using EZR software (Saitama Medical Center, Jichi Medical University, Japan) [18]. The threshold of significance was set at p <0.05.

## Results

Our results confirmed that there was no significant difference in blood glucose levels between the control and diabetic groups (119.1 ± 20.0 mg/dL) before streptozotocin administration (control group: 121.6 ± 12.5 mg/dL, diabetic group: 119.1 ± 20.0 mg/dL, p = 0.55). At 72 h after administration, all animals in the diabetic group had blood glucose levels of 300 mg/d or higher and were diagnosed with diabetes mellitus. Blood glucose levels in the control group were maintained in the normal range (117.2 ± 13.8 mg/dL). Body weights before streptozotocin administration were not significantly different between the control and diabetic groups (control group: 521.6 ± 12.1 g, diabetic group: 508.2 ± 40.5 g, p = 0.54). Significant weight loss was observed only in the diabetic group at eight and 16 weeks after induction (control group: 602.8 ± 26.7 g, diabetic group: 458.4 ± 85.0 g, p = 0.03; control group: 694.6 ± 48.7 g, diabetic group: 403.2 ± 40.2 g, p < 0.01).

Transcutaneous AGEs fluorescence measurement

**Figure 1:**
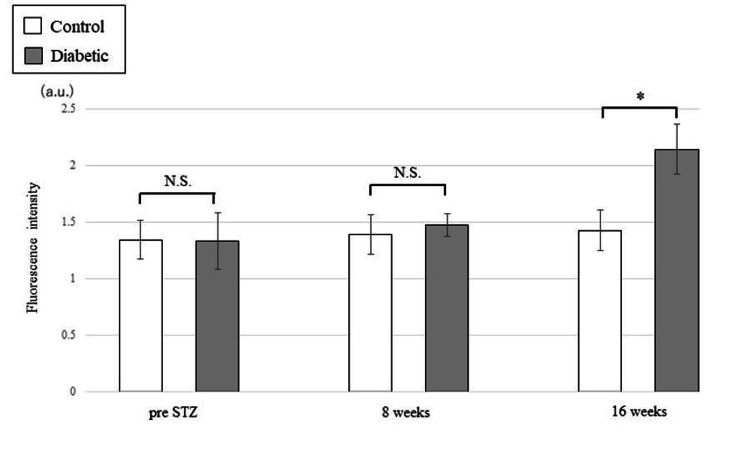
Changes in percutaneous fluorescence intensity of the auricle skin after streptozocin (STZ) administration The data are presented as the means ± SD.  *: p < 0.05 vs. Control (Mann-Whitney U test).

Transcutaneous fluorescence intensity measurements using auricular skin revealed no significant difference between the control and diabetic groups before streptozocin administration (control: 1.34 ± 0.17, diabetic: 1.33 ± 0.25, p = 0.65). At eight weeks after streptozocin administration, there was no significant difference between the control and diabetic groups (control: 1.39 ± 0.18, diabetic: 1.48 ± 0.10, p = 0.35). In contrast, the diabetic group exhibited a significant increase in fluorescence intensity compared with the control group after 16 weeks (control: 1.43 ± 0.18, diabetic: 2.14 ± 0.22, p = 0.01) (Figure 1).

Immunohistochemical findings

**Figure 2:**
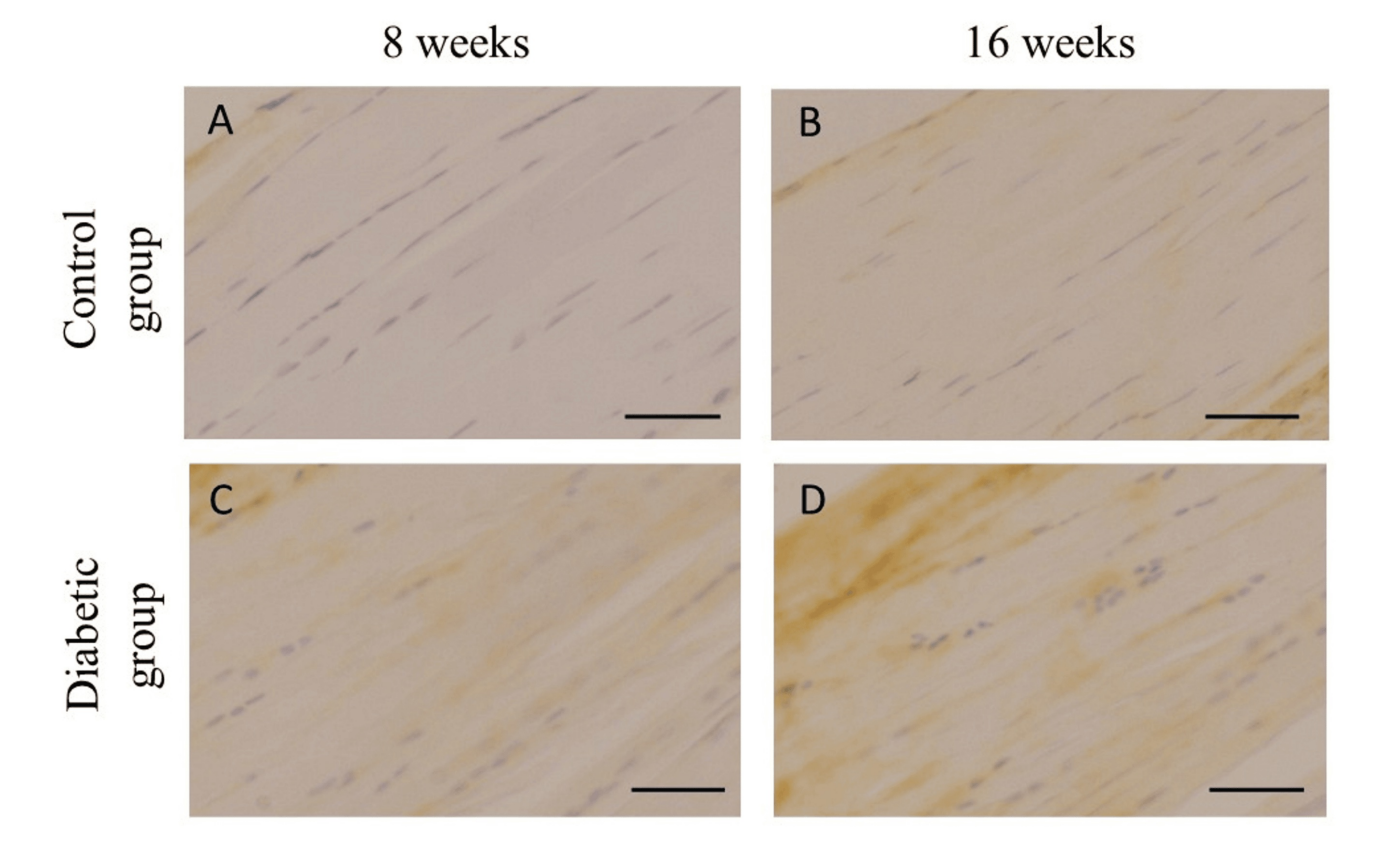
Immunohistochemical expression of advanced glycation end products (AGEs) Immunohistochemical expression of advanced glycation end products (AGEs) in control (A, B) and diabetic (C, D) rats at eight and 16 weeks after STZ administration. Scale bars = 50 µm.

All groups exhibited immunohistochemical expression of AGEs at the attachment site of the rotator cuff (Figure 2).

No statistically significant difference was observed in the area of AGEs staining in the diabetic group when compared with the control at eight weeks after streptozocin administration, although there was a trend toward a higher percentage in the diabetic group (control: mean 28.3±3.4%, diabetes: mean 32.6±3.8%, p = 0.12). At 16 weeks after administration, the diabetic group exhibited significantly enhanced immunohistological expression of AGEs at the rotator cuff attachment site compared with the control group (control: mean 30.1 ± 5.6%, diabetes: mean 36.8 ± 4.0%, p = 0.03) (Table 2).

**Table 2:** Quantitative immunohistochemistry in supraspinatus tendons after streptozocin (STZ) administration Values are expressed as mean ± standard deviation (SD). *: p < 0.05 vs. Control (Mann-Whitney U test) AGE: Advanced glycation end-products

STZ administration	After 8 weeks		After 16 weeks	
	Control (n=5)	Diabetic (n=5)	Control (n=5)	Diabetic (n=5)
AGEs (%)	28.3±3.4	32.6±3.8	30.1±5.6	36.8±4.0*
Collagen 1 (%)	68.9±7.1	67.9±9.7	66.3±6.2	69.3±8.2
Normal IgG (%)	7.6±3.8	7.3±3.3	7.1±2.4	7.8±3.3

**Figure 3:**
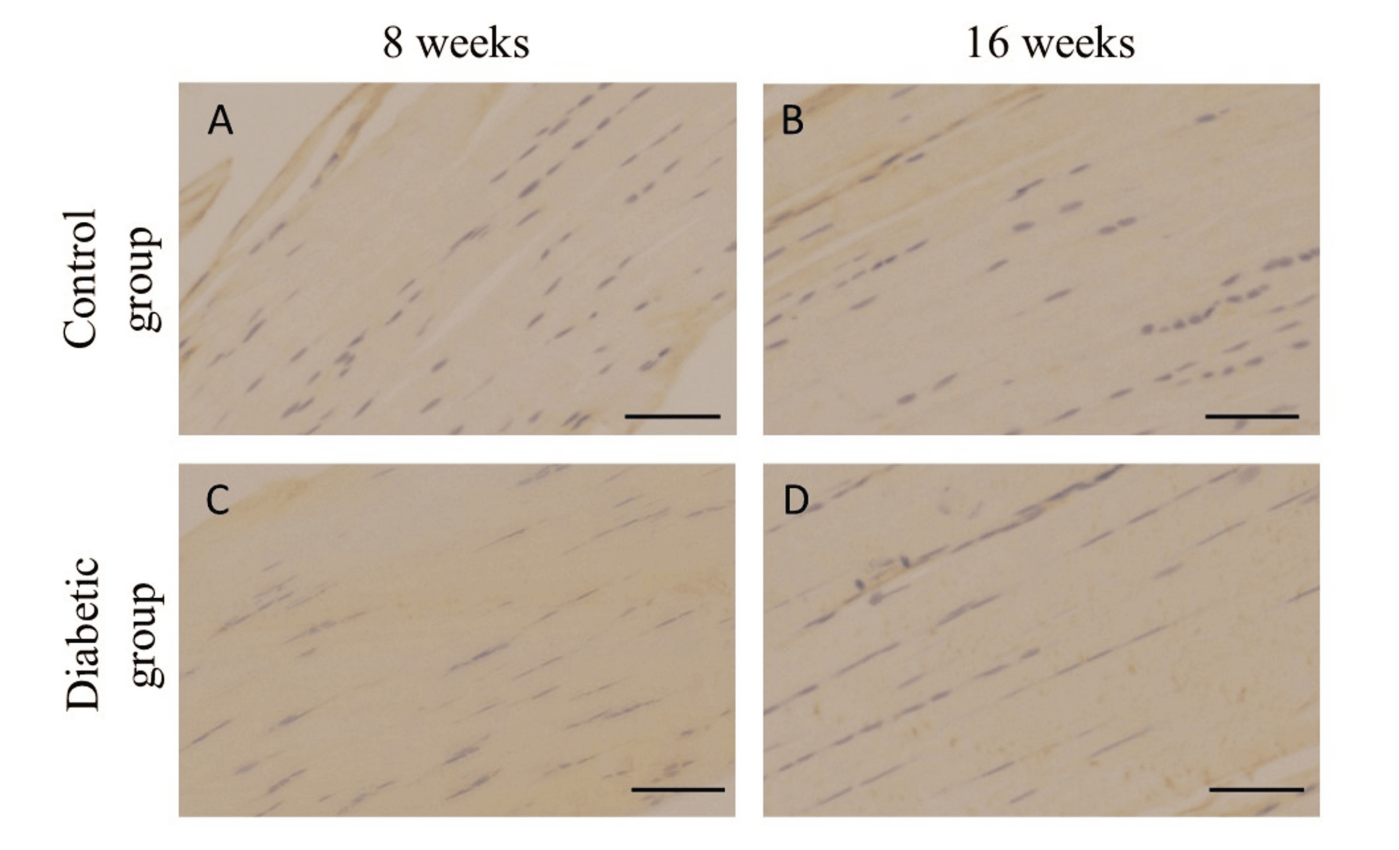
Immunohistochemical expression of Collagen I Collagen I is hardly stained on the rotator cuff attachment site in control (A, B) and diabetic rats (C, D). Scale bars = 50 µm.

**Figure 4:**
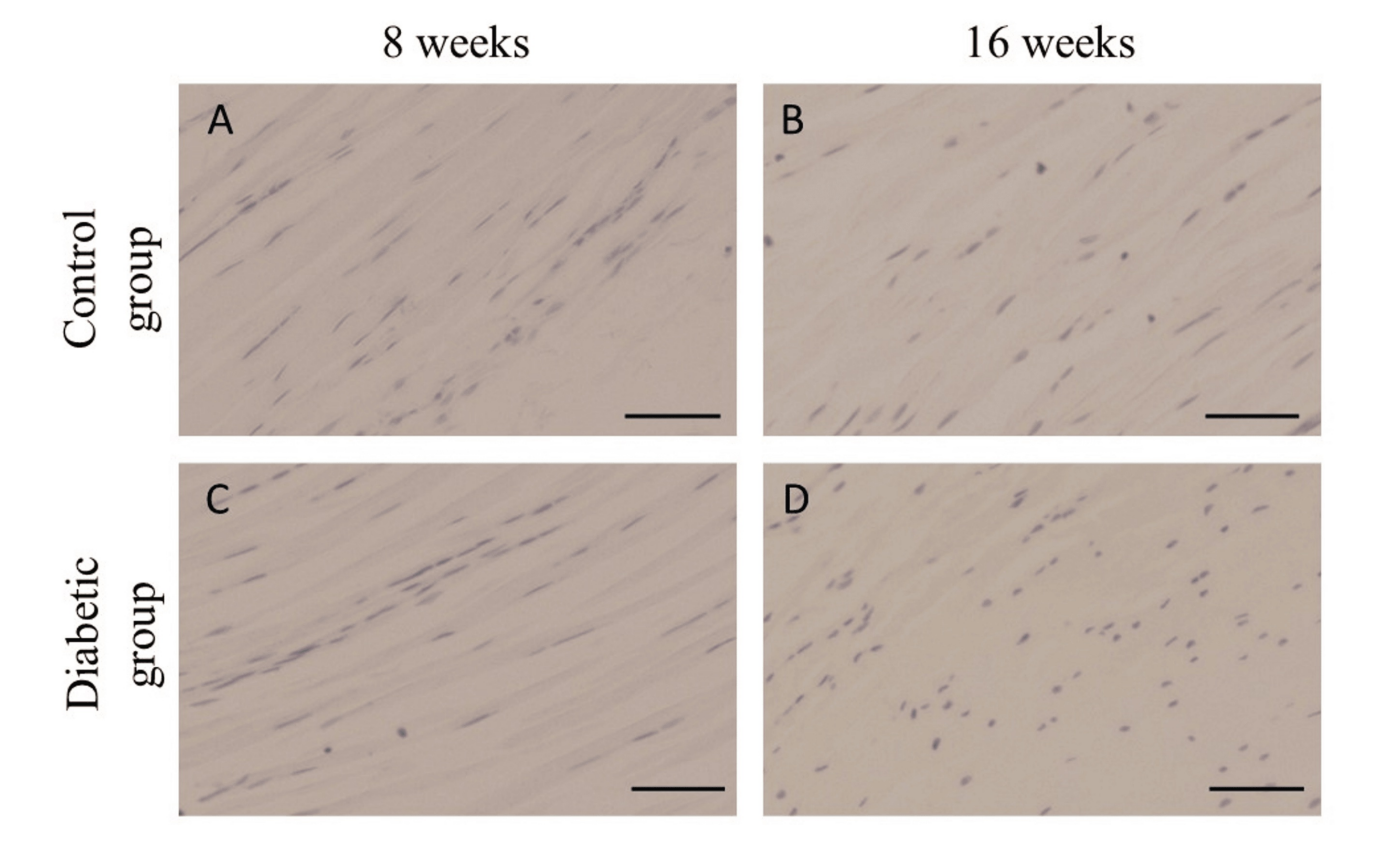
Immunohistochemical expression of Normal IgG Rotator cuff attachment site was not stained with normal IgG in control (A, B) and diabetic (C, D) rats. Scale bars = 50 µm.

**Figure 5:**
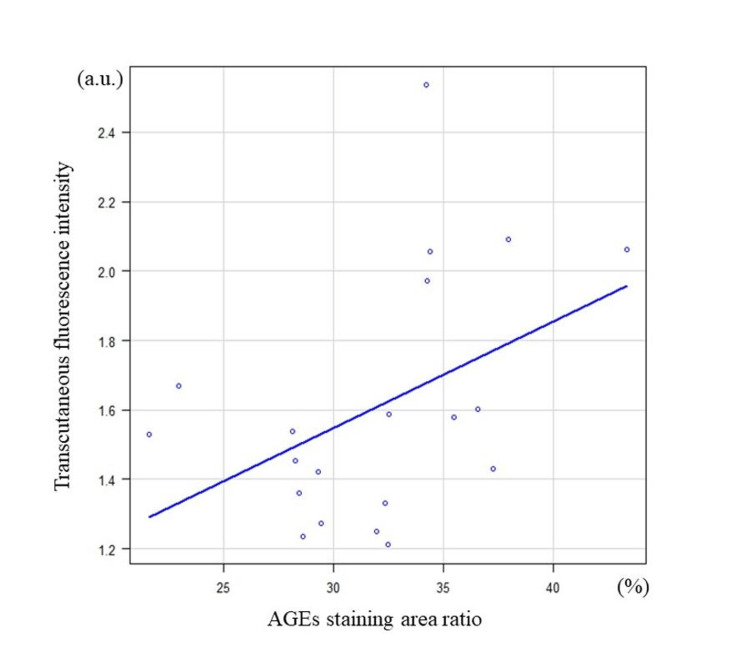
Relationship between transcutaneous fluorescence intensity and percentage of AGEs staining area r = 0.46, p = 0.02 (Spearman’s rank correlation coefficient)

No significant differences in the immunohistochemical expressions of collagen I and IgG were observed between the groups (Figures 3, 4); however, there was a positive correlation between transcutaneous fluorescence intensity measurements and the AGEs staining area ratio (Figure 5).

## Discussion

In this study, we evaluated the transcutaneous fluorescence intensity in rat auricular tissue and the accumulation of AGEs in rotator cuff tissue using a rat diabetes mellitus model. Our data demonstrated increased AGEs deposition in the rotator cuff area due to prolonged hyperglycemia caused by diabetes mellitus, and a positive correlation between histological pathology and transcutaneous fluorescence intensity. To our knowledge, this study is the first to reveal the association between non-invasively measured transcutaneous fluorescence intensity and histological AGEs accumulation in the rotator cuff. This novel finding introduces a potentially transformative method for assessing oxidative stress in rotator cuff tissue, particularly in diabetic patients.

AGEs are a by-product of the Maillard reaction produced in vivo [19], and their accumulation in tissues is known to activate inflammatory signals, causing chronic inflammation localized in collagen tissue [20,21]. Thus far, the measurement of AGEs in tissue samples has not been applied in clinical practice because it requires invasive procedures and multiple preparation steps. In this study, we created a diabetic model by administering streptozocin to SD rats and observed the accumulation of AGEs in rotator cuff tissue. We found that the accumulation of AGEs in the rotator cuff was heterogeneous and unevenly distributed at the rotator cuff attachment site. This suggests that degenerative rotator cuff injuries due to the accumulation of AGEs may occur at the cuff attachment site.

Previous studies have shown that patients with diabetes are at a higher risk for rotator cuff tears and are more likely to be hospitalized for repair surgery [4,22]. Moreover, patients with diabetes have a higher risk of re-tears after rotator cuff repair [23]. Histologically, it has also been reported that the formation and accumulation of AGEs is increased due to oxidative stress due to chronic hyperglycemia, and the extent of this stress correlates with the duration and severity of hyperglycemia and the presence of long-term complications [24,25]. Regarding the effects on rotator cuff tissue, increased AGEs accumulation in supraspinatus tendons has been reported [7]. In this study, immunohistochemical staining revealed that the AGEs-stained area at the supraspinatus muscle attachment site was not significantly different between diabetic and non-diabetic rats eight weeks after streptozotocin administration; however, a significant increase was observed in diabetic rats at 16 weeks. This suggests that the effects of oxidative stress on rotator cuff tissues appear to occur with long-term maintenance of hyperglycemia and an increased risk of rotator cuff rupture and postoperative re-tear.

Previous studies have shown that transcutaneous skin autofluorescence intensity can be measured non-invasively and rapidly and is associated with dermal AGEs accumulation [8,16]. Skin autofluorescence intensity is increased in patients with diabetes and has been shown to be associated with risk factors for long-term diabetic complications due to poor glycemic control and other factors [26]. It is also known to correspond to cardiovascular risk, as it has been reported to be related to carotid intima-media thickening and coronary artery calcification scores [27-29]. Yamanaka et al. reported that transcutaneous fluorescence intensity measurements can be used to predict diabetic microvascular complications [30]. In the transcutaneous evaluation in this study, there was a significant increase in transcutaneous auricular skin fluorescence intensity in diabetic rats at 16 weeks. Histological accumulation of AGEs exhibited a similar trend in the rotator cuff in the diabetic state, and a positive correlation was observed between dermal transdermal fluorescence intensity and the percentage of AGEs staining area. This suggests that it is possible to infer the accumulation of AGEs in rotator cuff tissue non-invasively using transcutaneous fluorescence intensity. It may also be used to predict the risk of AGEs-induced degeneration of rotator cuff tissue due to chronic inflammation and development of non-traumatic rotator cuff injuries.

Furthermore, for patients with suspected rotator cuff injuries or those undergoing surgery for rotator cuff tears, transcutaneous skin fluorescence intensity measurements, in addition to magnetic resonance imaging (MRI) and ultrasound, which are widely used today, could potentially provide information on tissue quality and assess the preoperative risk of possible postoperative re-tears after repair, as well as the risk of developing rotator cuff injuries. This may help physicians to consider a more appropriate fixation method at the time of surgery for high-risk populations.

This study has several limitations, including the small sample size, anatomical differences between human and rat shoulder joints, and the lack of mechanical strength assessments. The smaller size and differing biomechanical properties of the rat rotator cuff compared to humans may lead to variations in AGEs accumulation patterns and their impact on tendon function. Additionally, this study focused solely on type I diabetes induced by high doses of streptozotocin, which limits the generalizability of our findings to patients with type II diabetes. Further research is needed to explore the effects of oxidative stress on rotator cuff tissue in patients with type II diabetes mellitus.

## Conclusions

In conclusion, the results of this study suggest that transcutaneous fluorescence intensity could be a promising tool for assessing oxidative stress in the rotator cuff. However, given the limitations of the animal model used and the absence of mechanical strength assessments, these findings should be interpreted with caution. Further studies, particularly in human subjects and in models of type II diabetes, are necessary to validate the clinical utility of this method and to explore its potential role in predicting rotator cuff tear.
